# Personalized Antidepressant Selection and Pathway to Novel Treatments: Clinical Utility of Targeting Inflammation

**DOI:** 10.3390/ijms19010233

**Published:** 2018-01-12

**Authors:** Manish K. Jha, Madhukar H. Trivedi

**Affiliations:** Center for Depression Research and Clinical Care, UT Southwestern Medical Center, Dallas, TX 75390, USA; madhukar.trivedi@utsouthwestern.edu

**Keywords:** depression, antidepressants, inflammation, c-reactive protein, monoamines, monoclonal antibodies, experimental drugs, blood-brain barrier

## Abstract

Major depressive disorder (MDD) is a chronic condition that affects one in six adults in the US during their lifetime. The current practice of antidepressant medication prescription is a trial-and-error process. Additionally, over a third of patients with MDD fail to respond to two or more antidepressant treatments. There are no valid clinical markers to personalize currently available antidepressant medications, all of which have similar mechanisms targeting monoamine neurotransmission. The goal of this review is to summarize the recent findings of immune dysfunction in patients with MDD, the utility of inflammatory markers to personalize treatment selection, and the potential of targeting inflammation to develop novel antidepressant treatments. To personalize antidepressant prescription, a c-reactive protein (CRP)-matched treatment assignment can be rapidly implemented in clinical practice with point-of-care fingerstick tests. With this approach, 4.5 patients need to be treated for 1 additional remission as compared to a CRP-mismatched treatment assignment. Anti-cytokine treatments may be effective as novel antidepressants. Monoclonal antibodies against proinflammatory cytokines, such as interleukin 6, interleukin 17, and tumor necrosis factor α, have demonstrated antidepressant effects in patients with chronic inflammatory conditions who report significant depressive symptoms. Additional novel antidepressant strategies targeting inflammation include pharmaceutical agents that block the effect of systemic inflammation on the central nervous system. In conclusion, inflammatory markers offer the potential not only to personalize antidepressant prescription but also to guide the development of novel mechanistically-guided antidepressant treatments.

## 1. Introduction

Major depressive disorder (MDD) affects one in six adults in the United States during their lifetime [1,2]. For most patients, MDD has a chronic course that is marked either by persistent symptoms or by repeated depressive episodes interspersed with periods of symptomatic improvement [3]. Additionally, patients with MDD report significant impairment across multiple domains of life such as work productivity [4], non-work-related day to day activities [5], psychosocial function [6], and quality of life [7]. The disability associated with MDD has increased over 50% in the last two decades, making it the second leading cause of global disability [8]. The economic burden of MDD is estimated to exceed $200 billion per year [9]. To reduce the disability and economic burden associated with MDD and to improve the poor clinical outcomes in clinical practice [10], there is an urgent need to parse through the syndromic and etiological heterogeneity of MDD [11]. This report aims to briefly review the role of inflammation in the pathophysiology of depression along with the recent findings of inflammatory markers moderating antidepressant treatment outcomes and the efficacy of monoclonal antibodies in reducing depressive symptoms in patients with chronic inflammatory conditions. Additionally, a theoretical framework and potential future studies are discussed in order to personalize the prescription of currently available antidepressants and to identify novel treatments.

## 2. Need for Personalized Antidepressant Prescription

The current practice guidelines recommend prescribing antidepressant medications based either on clinical characteristics (such as side-effect profile, previous history of response) or non-clinical factors (such as patient or provider preference, cost, availability on insurer’s approved drug list) [12]. This is despite a lack of any clinical evidence that such clinical or non-clinical factors can guide antidepressant medication prescription. Clinical factors such as severity of depressive symptoms at baseline [13], onset of MDD before the age of 18 year [14], persistence of index major depressive episode longer than 2 years [15] or the presence of insomnia prior to treatment initiation [16] did not moderate outcomes in reports that compared selective serotonin reuptake inhibitor (SSRI) monotherapy with antidepressant medication combinations. Similarly, commonly used clinical subtypes of depression defined by the presence of atypical, melancholic or anxious features have failed to predict any significant difference among currently available antidepressant medications [17,18]. This is consistent with the failure to find any significant differences in head-to-head trials of antidepressant medications within or across different classes [19]. Hence, the current clinical practice continues to be a trial-and-error process that necessitates multiple treatment trials to attain adequate symptomatic control for a majority of patients [20,21]. Unsurprisingly, most patients stay on ineffective medications for too long, switch treatments too early, or simply drop out of care [22,23]. Thus, there is an urgent need to personalize antidepressant treatment by maximizing the likelihood of improvement and minimizing the risk of adverse events [24].

## 3. Need for Novel Antidepressants

Large community trials such as the Sequenced Treatment Alternatives to Relieve Depression (STAR*D) trial have shown that less than a third of patients adequately respond to the initial antidepressant medication trial [25]. In fact, over 35% of depressed patients fail to respond to two or more adequate courses of antidepressant medications [20,21,26], i.e., they have treatment-resistant depression (TRD). These patients experience persistence of depressive symptoms over a long period of time and are exposed to adverse consequences of ineffective medications. Additionally, TRD patients report higher rates of suicidality and lower quality of life as compared to treatment responsive depressed patients [27]. All currently available and commonly prescribed antidepressant medications target monoamine neurotransmission [28,29,30]. Thus, there is an urgent need to identify antidepressant medications with novel non-monoaminergic mechanisms of action. The search for novel antidepressants is currently hindered by our subjective practice of diagnosing depressive disorder. The National Institute of Mental Health has launched the Research Domain Criteria (RDoC) initiative to promote novel objective ways for conceptualizing and classifying mental disorders [31]. The RDoC initiative offers an opportunity to understand the pathophysiology of depressive disorders. Novel antidepressants that are target-driven against the pathophysiological mechanisms offer the potential for personalized medicine for MDD patients.

## 4. Role of Inflammation in Depression

Several lines of investigation implicate inflammation in the pathophysiology of depression in a sub-group of patients with MDD [32,33]. Patients, who receive cytokines as a treatment for their medical conditions, such as hepatitis C or malignancies, develop MDD at high rates. Over a third of patients initiated on interferon α (IFN-α) treatment for chronic hepatitis C develop moderate or severe depressive symptoms [34]. While IFN-α results in worsening of symptoms across multiple domains of MDD (mood, cognition, anxiety, and neurovegetative), treatment with antidepressant medication results only in the improvement of core mood and not in other symptom domains [35]. Additionally, patients who developed depressive symptoms following IFN-α treatment had poorer hepatitis C viral clearance as compared to those who did not develop depressive symptoms [36]. Due to recent advances in the treatment of hepatitis C, the use of IFN-α has decreased markedly [37], thus restricting the utility of this treatment paradigm to study the role of immune dysfunction in depression.

Patients with MDD also have elevated markers of non-specific inflammation, such as c-reactive protein (CRP). Produced by the liver, CRP is a pentameric protein that increases thousands of folds in response to acute infection or injury and is hence also referred to as acute-phase reactant [38]. Chronic low-grade systemic inflammation is associated with elevated CRP levels, which in turn have been associated with increased mortality (both vascular and non-vascular) and a higher likelihood of cardiovascular disease and ischemic stroke [39]. In depressed patients, elevated CRP levels have been associated with higher likelihood of hospitalization related to depression [40]. In epidemiologic studies, higher levels of CRP have been associated with higher severity of depressive symptoms [41]. In a recent report from the Genome-Based Therapeutics Drugs for Depression (GENDEP) study, the association of CRP with depression severity was seen only in women but not men [42]. This may have been related to the higher proportion of females in the GENDEP study as compared to males. Notably, other studies have reported an association of CRP with depression only in males and not in females [43]. In addition to the association with depression severity and risk of inpatient hospitalization, high levels of CRP have also been associated with greater likelihood of completed suicide [44].

Among specific inflammatory markers, elevated levels of interleukin 6 (IL-6) have been most consistently reported in patients with MDD as compared to healthy controls. Several meta-analyses have found elevated levels of IL-6 in peripheral circulation in depressed patients as compared to controls with moderate to large effect size [45,46,47,48]. Higher levels of IL-6 have also been reported in cerebrospinal fluid of depressed patients [49] as well as in suicide attempters [50] as compared to non-depressed controls. Higher levels of both CRP and IL-6 have also been shown to predict subsequent depressive symptoms [51]. Obesity partly accounts for the elevated IL-6 levels in depressed patients [52]. Patients with MDD also have elevated levels of other pro-inflammatory cytokines such as tumor necrosis factor α (TNF-α).

Recent evidence also implicates interleukin 17 (IL-17) in the pathophysiology of MDD. One of the downstream consequences of IL-6 elevation is the differentiation of native T-helper (Th) lymphocytes into IL-17 producing Th17 lymphocytes, thus promoting secretion of IL-17 [53]. The role of IL-17, which was initially identified in 1995 [54], and Th17 cells, which were identified as distinct from the common Th1 and Th2 sub-types in 2005 [55,56], is well established in the pathophysiology of systemic inflammatory disorders such as psoriasis, systemic lupus erythematosus, asthma, and rheumatoid arthritis [57]. In an elegant set of animal experiments, Beurel et al. recently demonstrated the role of IL-17 and Th17 cells in the pathophysiology of depression [58]. They showed that (1) levels of Th17 cells were higher in brains of rodents that exhibited learned helplessness; (2) infusion of Th17 cells was associated with depression-like behaviors at sub-threshold stimulation; (3) infusion of anti-IL-17 antibody or administration of SR1001, an inhibitor of retinoid-related orphan receptor- γT (RORγT, a transcription factor essential for differentiation of naïve CD4+ T cells to Th17 cells) mitigated the effects of Th17 cell infusion; and (4) RORγT knockout mice exhibited marked resistance to learned helplessness paradigm [58]. While one study has previously reported higher levels of Th17 cells and lower regulatory T cells in peripheral circulation, along with higher levels of RORγT mRNA in peripheral blood lymphocytes of depressed patients as compared to control subjects [59], a recent meta-analysis did not find a significant difference in IL-17 levels between depressed and healthy control subjects [47].

Chemokines have been implicated in depression by facilitating migration of peripheral immune cells into the central nervous system [60]. A recent meta-analysis found elevated chemokines (CXCL8, and CXCL 7) and reduced levels of CCL4 in plasma of depressed patients as compared to healthy controls, of which plasma CXCL8 had a negative predictive value of 93.5% [61].

## 5. Pathophysiological Mechanisms Underlying Role of Inflammation in Depression

While the brain has been considered an immune-privileged organ, emerging evidence implicates the role of peripheral inflammation in the pathophysiology of depression [62]. The factors that increase the likelihood of inflammation in depression have been reviewed in detail by Kiecolt-Glaser et al. [63]. Peripheral injection of lipopolysaccharide (LPS) in rodents is associated with depressive symptoms, including anhedonia, even after the acute sickness syndrome has resolved and serves as a model to demonstrate the pathophysiological role of inflammation in depression [32]. In humans, injection of LPS is associated with worsening of depressive symptoms and reduction in ventrostriatal reward activity to monetary reward cues [64]. Peripheral inflammation leads to activation of indoleamine oxygenase (IDO), which diverts tryptophan metabolism from serotonin to kynurenine [32]. Peripheral kynurenine is transported across the blood-brain barrier (BBB) by the L-type amino acid transporter (LAT-1) and is converted to quinolinic acid in microglia by activation of kynurenine 2-monooxygenase (KMO) [65]. An additional mechanism underlying the role of inflammatory cytokines in depression is related to the susceptibility to stress. In rodent studies of repeated social defeat stress (RSDS), animals susceptible to stress exhibited increased levels of IL-6 early after exposure to stress as compared to those who were resistant to stress [66].

The putative mechanisms underlying the role of IL-17 involves its effect on the BBB. Peripheral IL-17 binds to the IL-17 receptors on the BBB leading to the generation of reactive oxygen species (ROS), which in turn increase BBB permeability [67]. Increased BBB permeability is associated with infiltration of immune cells, which in turn have been shown to promote depressive behavior [60,68]. Dysfunction of the BBB induces nitric oxide synthase (NOS) and produces inflammatory cytokines from microglial cells [69,70]. The resultant neuroinflammation diverts tetrahydrobiopterin, an essential cofactor of both NOS and tyrosine hydroxylase, away from the conversion of tyrosine to l-3,4-dihydroxyphenylalanine, the rate-limiting step of dopamine synthesis [71]. Reduced dopamine synthesis, in turn, is associated with worsening of symptoms of anhedonia [64,72,73,74]. Thus, peripheral inflammation has been shown to affect serotonin, dopamine, and glutamate neurotransmitter systems.

## 6. Effects of Antidepressant Treatments on Inflammation

It is widely acknowledged that antidepressant treatments affect the immune system. A recent meta-analysis found significant reductions in IL-4, IL-6, IL-1β (specific only to SSRIs) and IL-10 after antidepressant treatment along with no changes in IL-2, TNF-α, IFN-γ, and CRP [75]. These findings were partly replicated in a difference meta-analysis, which reported reductions in IL-6, IL-10, TNF-α, and CCL-2 with antidepressant treatment along with no changes in IL-1β, IL-2, and IFN-γ [76]. In a rodent model, administration of citalopram, an SSRI medication, was associated with increased levels of proinflammatory cytokines (IL-1β, IL-6, TNF-α, and IFN-γ) in the frontal cortex, and co-administration of non-steroidal anti-inflammatory drugs (NSAIDs) blocked the increase of cytokines but also resulted in the loss of effect of citalopram in animal models of depression [77]. A potential reason for this increase in Th1-related cytokines by SSRIs could be their effect on Th2 cell-mediated immune response. SSRIs have been shown to suppress IL-2 and IL-4-producing cells in the thymus [78]. Reviews of the effect of antidepressant medications on cytokine levels have been more mixed. Most studies demonstrate a decrease in IL-6 with antidepressant treatment [79], which is not correlated with a reduction in depression severity [80] and is most notable specifically with SSRI medications [81]. Among other inflammatory cytokines, persistently elevated levels of TNF-α have been associated with a failure to respond to antidepressant medications [80]. In a recent report, Gadad et al. found that an increase in eotaxin levels after antidepressant treatment was associated with improved treatment outcomes, whereas a decrease in IFN-γ was associated with failure to remit after 12 weeks of antidepressant treatment [82]. Based partly on these findings, Martino et al. proposed a theoretical model whereby serotonergic antidepressants suppress the Th2-mediated immune response, whereas noradrenergic antidepressants suppress the Th1-mediated immune response [83]. However, this model is limited by the omission of innate immune markers, Th17 cell-mediated immune response, and the antidepressant effect of dopaminergic medications as well as anti-inflammatory medications.

Bupropion, a dopaminergic noradrenergic antidepressant, has been shown to suppress the Th1- [84] and Th17- [85] mediated immune response. Similarly, pramipexole, a dopamine agonist with evidence of efficacy in treatment-resistant depression [86], has been shown to inhibit the production of IL-17 [87]. Exercise, an effective augmentation strategy after initial non-response to SSRI medication [88], has been shown to be more effective in depressed patients with higher levels of TNF-α at baseline [89]. Additionally, reduction in IL-1β with exercise is positively correlated with a reduction in overall depression severity and hypersomnia [89,90].

## 7. Inflammatory Markers to Personalize Antidepressant Prescription

**Figure 1:**
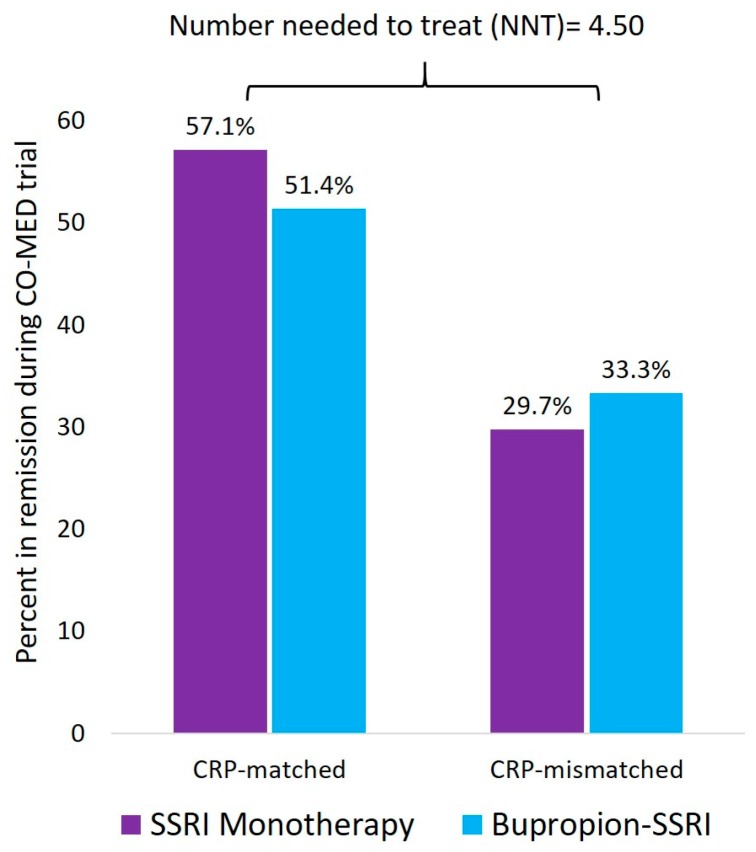
Superiority of CRP-matched treatment assignment to SSRI monotherapy of combination of bupropion and SSRI. CRP is c-reactive protein, CO-MED is Combining Medications to Enhance Depression Outcomes, SSRI is selective serotonin reuptake inhibitor. This figure is based on the findings reported by Jha et al. [92]. CRP-matched treatment assignment refers to participants who received escitalopram only and had CRP < 1 mg/L received escitalopram whereas those with CRP ≥ 1 mg/L received bupropion-SSRI combination. The rest of the participants were grouped in the CRP-mismatched category. The NNT was obtained by subtracting the remission rate in the CRP-mismatched assignment (30.9%) from the remission rate in the CRP-matched assignment (53.1%) and dividing the aforementioned difference by 100.

Inflammatory biomarkers are poised for widespread application to profoundly change the current clinical practice. Systematic reviews have found that elevated inflammation predicts poor response to commonly used antidepressant medications [80]. Two recent reports have shown CRP, a non-specific marker of inflammation can help in selecting between antidepressants with serotonergic versus non-serotonergic action. In the initial report, Uher et al. used data from the GENDEP study to evaluate if CRP at baseline predicted differential reduction in depression severity with escitalopram versus nortriptyline [91]. They found that depressed patients with CRP levels less than 1 mg/L prior to treatment initiation experienced significantly greater reduction in depression severity with escitalopram as compared to nortriptyline. Conversely, depressed patients with CRP levels ≥ 1 mg/L responded significantly better to nortriptyline as compared to escitalopram. In an unrelated study, Jha et al. recently evaluated if baseline CRP levels predicted differential response to escitalopram monotherapy versus bupropion-escitalopram combination [92]. Depressed patients with lower CRP levels responded better to escitalopram monotherapy whereas those with higher levels responded better to bupropion escitalopram combination. Depressed patients with biomarker matched treatment (those with CRP < 1 mg/L received escitalopram whereas those with CRP ≥ 1 mg/L received bupropion-SSRI combination) had significantly higher remission rates (53.1%) as compared to the 30.9% remission rate in the biomarker mismatched treatment arm. As shown in Figure 1, this potential CRP-matched treatment assignment had a number-needed-to-treat (NNT) = 4.5. Additionally, it is worthwhile to note that the remission rate with the CRP-matched treatment assignment was significantly higher than that with the first step treatment in the STAR*D study where only 33% depressed patients attained remission with citalopram monotherapy [25].

While CRP is a clinically pragmatic biomarker for treatment assignment, its level may be elevated due to a multitude of acute and chronic factors. Thus, there is a need to identify more specific factors which can guide differential treatment selection among currently available antidepressant medications. Among specific inflammatory markers, IL-17 has emerged as a potential candidate. In two separate samples, Hennings et al. recently demonstrated that lower pre-treatment levels of ROR α mRNA, a transcription factor involved in differentiation of naïve CD4+ T cells into Th17 cells [57], were associated with better response to antidepressant treatment [93]. Hence, Jha et al. recently explored a panel of IL-17, Th1- (IFN-γ and TNF-α), Th2- (Il-4, IL-5, IL-9, and IL-13), and non-T cell-related (IL-1β, IL-1 receptor antagonist, IL-6, IL-8, and macrophage inflammatory protein (MIP) 1 α and β) markers as antidepressant treatment selection biomarkers. While depressed patients with elevated IL-17 levels prior to treatment initiation experienced a greater reduction in depression severity with the bupropion-SSRI combination as compared to those with lower IL-17 levels, no such association was seen in SSRI monotherapy and venlafaxine-mirtazapine combination treatment arms [94]. In a follow-up report that evaluated the role of platelet-derived growth factor (PDGF), Jha et al. reported that improvement in anhedonia completely accounted for the differential improvement in depression severity seen with bupropion-SSRI combination versus SSRI monotherapy based on PDGF levels [95].

## 8. Anti-Inflammatory Drugs as Novel Antidepressants

While personalizing the prescription of currently available antidepressant medications can improve clinical outcomes for over half the patients with MDD in 12 weeks [86], novel antidepressants are still needed for those patients with TRD. The potential antidepressant effect of anti-inflammatory drugs is suggested by the efficacy of NSAIDs, specifically celecoxib, as an adjunctive treatment in patients with MDD [96]. Anti-cytokine treatments have emerged as candidates for novel antidepressants. They offer the potential to specifically target inflammatory pathways that have been implicated in the pathophysiology of depression. In the first study of its kind, Raison et al. recruited 60 TRD patients with no history of systemic inflammatory disorders and randomized them to either placebo or infliximab, a monoclonal antibody against TNF-α. While there was no overall difference between infliximab and placebo, in post hoc analyses they found that in a subgroup of TRD patients with CRP ≥ 5 mg/L, infliximab was superior to the placebo in the improvement of depressive symptom severity [97]. Husain et al. recently found a significant antidepressant effect in a meta-analysis of anti-inflammatory agents (including adjunctive NSAIDs, infliximab, and minocycline) in patients with MDD [98]. In another recent meta-analysis, Kappelmann et al. reported significant improvement in depressive symptoms with anti-cytokine treatments in patients with chronic inflammatory conditions [99]. While agents against TNF-α have been studied most often, humanized monoclonal antibody against IL-6 has also been shown to be effective in reduction of depressive symptoms [100]. Two recent reports have also raised the antidepressant potential of monoclonal antibodies targeting IL-17-mediated immune response. Specifically, a phase 3 trial of brodalumab, a monoclonal antibody against the IL-17 receptor, evaluated its effect on depressive symptoms in psoriasis patients who had moderate/severe depression at baseline (*n* = 106). The rates of symptomatic remission (improved to normal on the hospital anxiety and depression rating scale) were significantly higher (*p* < 0.05) with brodalumab 140mg q2week (47%) and 210 mg q2week (43%) as compared to the placebo (9%) [101]. Notably, improvement in psoriasis symptoms did not completely account for the improvements in depressive symptom severity. A similar improvement in depression has also been reported with ixekizumab, a monoclonal antibody against IL-17. In a recent report based on three double-blind randomized controlled phase 3 trials, Griffiths et al. reported on psoriasis patients with moderate or severe depression severity (*n* = 320), defined as scores ≥ 11 on the Quick Inventory of Depressive Symptomatology Self-Report [102]. Remission rates after 12 weeks of ixekizumab treatment at 80 mg every 4 weeks and 80 mg every 2 weeks were 33.6% and 45.2%, i.e., significantly higher (*p* < 0.01) than the remission rate of 17.8% with placebo [102]. However, these anti-IL-17 treatments have not been studied in depressed patients without autoimmune diseases. Non-targeted treatment with anti-cytokine treatment in depressed patients carries substantial risk. Further caution with the use of anti-IL-17 treatment is warranted due to reports of 2 completed suicides during phase 3 trials of brodalumab [103,104,105].

## 9. Future Directions

Future studies are needed to test the superiority of inflammatory marker-based antidepressant prescription relative to the current practice of clinical decision-making. A CRP-matched treatment assignment offers the most pragmatic choice in this regard [106]. In individual patients, CRP levels are unaffected by time of the day or meal intake, varies little year-to-year in the absence of acute events, and can be measured inexpensively through commercial laboratories [38,107,108]. In fact, CRP levels can now be measured with a fingerstick to provide clinically-actionable information in a primary care setting [109,110]. Real-world clinical trials are needed to test if implementing a CRP level-based treatment assignment results in higher rates of remission as compared to high-quality measurement-based care. Further work is also needed to identify the pathophysiological mechanism which underlies the differential treatment responses seen with serotonergic versus non-serotonergic antidepressants. Finally, these findings have opened the potential to guide the selection of dopaminergic drugs in the treatment of depression—an exciting potential, especially for treatment-resistant depressed patients who have failed to respond to currently FDA approved antidepressants.

**Figure 2:**
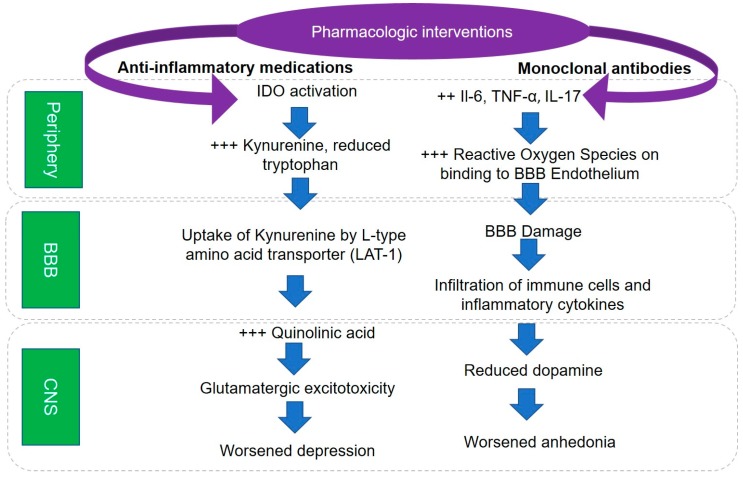
Theoretical framework for developing novel antidepressants by targeting inflammatory pathways. Two distinct pharmacologic interventions with the potential to reduce depressive symptom severity. In the first pathway, activation of indoleamine oxygenase (IDO) results in increased levels of kynurenine, which is taken up by LAT-1 transporters and converted to Quinolinic acid by microglial cells. This results in glutamatergic excitotoxicity and depressive symptoms. Blockade of the LAT-1 transporter by a pharmacologic agent can disrupt this cascade and reduce depressive symptoms and mitigate central nervous system (CNS) effects of peripheral inflammation. Similarly, anti-cytokine treatments may be effective in depressed patients with elevated levels of inflammatory cytokines (interleukin 6 or IL-6, interleukin 17 or IL-17, and tumor necrosis factor alpha or TNF-α), which result in blood-brain barrier (BBB) dysfunction.

In the search for novel antidepressants targeting inflammation, Figure 2 presents a theoretical framework. In the subset of depressed patients who exhibit elevated inflammatory cytokines, such as IL-6, IL-17, and TNF-α, targeted use of monoclonal antibodies against these cytokines can result in reduced anhedonia and overall depression severity. A major limitation of this approach is the lack of tests for these cytokines through commercial CLIA certified labs. However, it is noteworthy that an ongoing phase 2 clinical trial is testing the efficacy of augmentation with sirukumab, a monoclonal antibody against IL-6, in depressed patients with CRP levels ≥ 3 mg/L (NCT02473289) and in TRD patients who have failed to respond to at least one but no more than three adequate antidepressant treatments during their current episode of depression.

A parallel approach, as outlined by Remus et al., targets transport of kynurenine at the BBB to reduce the effect of peripheral inflammation on the central nervous system. This approach is based on the earlier work by Dantzer et al., which showed that peripheral inflammation leads to the induction of IDO, which in turn diverts tryptophan away from the synthesis of serotonin to that of kynurenine [65]. Kynurenine, in turn, is taken up the LAT-1 present on the BBB and converted to quinolinic acid in microglial cells by the induction of KMO. As l-leucine, an essential amino acid is also a substrate for the LAT-1 transporter, harmful effects of peripheral inflammation can be mitigated by oral administration of l-leucine. A pilot double-blind placebo-controlled study is currently underway to test this hypothesis (NCT03079297).

## 10. Conclusions

Due to the poor outcomes of depressed patients in clinical practice, there is a pressing need to identify newer treatments and better strategies to personalize currently available antidepressant treatments. In the search for biomarkers to personalize antidepressant medication selection, inflammatory biomarkers, such as CRP, have emerged as a robust and pragmatic option. However, as over a third of patients with MDD are resistant to currently available medications, novel antidepressants that target the pathophysiology underlying depression are also needed. Anti-cytokine treatments have emerged as potentially selective agents to target proinflammatory cytokines in those patients with markers of systemic inflammation. Additional strategies include targeting of the BBB to mitigate the CNS effects of peripheral inflammation. In conclusion, inflammatory markers present clinically useful targets for both personalizing antidepressant prescription and for identifying novel antidepressants.

## Abbreviations

MDD	Major depressive disorder
SSRI	selective serotonin reuptake inhibitor
STAR*D	Sequenced Treatment Alternatives to Relieve Depression
TRD	Treatment-Resistant Depression
RDoC	Research Domain Criteria
IFN-α	Interferon Alpha
CRP	C-Reactive Protein
GENDEP	Genome-Based Therapeutics Drugs for Depression
TNF-α	Tumor Necrosis Factor Alpha
IL-17	Interleukin 17
Th	T-helper
RORyT	Retinoid-related Orphan Receptor-yT
LPS	Lipopolysaccharide
IDO	Indoleamine Oxygenase
LAT-1	L-type Amino Acid Transporter
KMO	Kynurenine 2-Monooxygenase
BBB	Blood-Brain Barrier
ROS	Reactive Oxygen Species
NOS	Nitric Oxide Synthase
NSAIDs	Non-Steroidal Anti-Inflammatory Drugs
